# Zoonotic Transmission of *Campylobacter jejuni* to Caretakers From Sick Pen Calves Carrying a Mixed Population of Strains With and Without Guillain Barré Syndrome-Associated Lipooligosaccharide Loci

**DOI:** 10.3389/fmicb.2022.800269

**Published:** 2022-04-29

**Authors:** Jessica L. St. Charles, Phillip T. Brooks, Julia A. Bell, Husnain Ahmed, Mia Van Allen, Shannon D. Manning, Linda S. Mansfield

**Affiliations:** ^1^Comparative Enteric Diseases Laboratory, Department of Large Animal Clinical Sciences, College of Veterinary Medicine, Michigan State University, East Lansing, MI, United States; ^2^Comparative Medicine and Integrative Biology, College of Veterinary Medicine, Michigan State University, East Lansing, MI, United States; ^3^Institute for Integrative Toxicology, Michigan State University, East Lansing, MI, United States; ^4^College of Veterinary Medicine, Michigan State University, East Lansing, MI, United States; ^5^Department of Microbiology and Molecular Genetics, Michigan State University, East Lansing, MI, United States

**Keywords:** *Campylobacter jejuni*, Guillain Barré Syndrome, autoimmunity, gastrointestinal inflammation, zoonoses, outbreak investigation

## Abstract

*Campylobacter jejuni* causes foodborne gastroenteritis and may trigger acute autoimmune sequelae including Guillain Barré Syndrome. Onset of neuromuscular paralysis is associated with exposure to *C. jejuni* lipooligosaccharide (LOS) classes A, B, C, D, and E that mimic and evoke antibodies against gangliosides on myelin and axons of peripheral nerves. Family members managing a Michigan dairy operation reported recurring *C. jejuni* gastroenteritis. Because dairy cattle are known to shed *C. jejuni*, we hypothesized that calves in the sick pen were the source of human infections. Fecal samples obtained from twenty-five calves, one dog, and one asymptomatic family member were cultured for *Campylobacter*. *C. jejuni* isolates were obtained from thirteen calves and the family member: *C. coli* from two calves, and *C. hyointestinalis* from two calves. Some calves had diarrhea; most were clinically normal. Typing of lipooligosaccharide biosynthetic loci showed that eight calf *C. jejuni* isolates fell into classes A, B, and C. Two calf isolates and the human isolate possessed LOS class E, associated mainly with enteric disease and rarely with Guillain Barré Syndrome. Multi-locus sequence typing, *por*A and *fla*A typing, and whole genome comparisons of the thirteen *C. jejuni* isolates indicated that the three LOS class E strains that included the human isolate were closely related, indicating zoonotic transmission. Whole-genome comparisons revealed that isolates differed in virulence gene content, particularly in loci encoding biosynthesis of surface structures. Family members experienced diarrheal illness repeatedly over 2 years, yet none experienced GBS despite exposure to calves carrying invasive *C. jejuni* with LOS known to elicit antiganglioside autoantibodies.

## Introduction

*Campylobacter jejuni* is the leading cause of human bacterial gastrointestinal infection in the Western world (Sahin et al., 2002; Church Potter and Kaneene, 2003; Vegosen et al., 2012). It is estimated that 2.1–2.5 million cases of campylobacterosis occur annually in the U.S. (Fitzgerald et al., 2001). Symptoms of *C. jejuni* infection include fever, abdominal cramping, and watery or bloody diarrhea (Altekruse et al., 1999; Sahin et al., 2002). The majority of *C. jejuni* infections are the result of ingestion of undercooked chicken, but it has been shown that contact with household pets and the ingestion of raw milk, contaminated water, or undercooked beef or pork can lead to infection (Altekruse et al., 1999; Sahin et al., 2002). *Campylobacter* spp. are widely spread in agricultural environments; poultry and farm animals are natural reservoirs for *C. jejuni* (Fitzgerald et al., 2001; French et al., 2005; Sheppard et al., 2011b; Sproston et al., 2011; Mourkas et al., 2020). *C. jejuni* has been isolated from the intestines of chickens and found in the feces of domesticated household pets, beef cattle, and dairy cattle (Fitzgerald et al., 2001; Vegosen et al., 2012). *C. jejuni* has also been detected in bulk tank milk (Bianchini et al., 2014a).

Dairy products are associated with *C. jejuni* outbreaks, while poultry products are associated with sporadic infections (Jay-Russell et al., 2013). Data for 2012–2018 from the US Centers for Disease and Prevention National Outbreak Reporting System (Centers for Disease Control and Prevention, 2018) indicate that of 352 *Campylobacter* outbreaks in 2012 through 2018, 56 (16%) were listed as confirmed or suspected to be due to animal contact. Of these 56 outbreaks, thirty were associated with consumption of dairy or beef products; two were associated with mixed infections of *Campylobacter* with either *Giardia* or *Salmonella*. Three outbreaks were confirmed to have been associated with consumption of unpasteurized milk (Centers for Disease Control and Prevention, 2018). *C. jejuni* has been detected in bulk tank milk on a farm producing raw milk for human consumption (Bianchini et al., 2014a).

A Swiss study examined 395 calves from cow-calf farms for enteropathogenic bacteria and showed that *C. jejuni* was present in 32% of the calves (Busato et al., 1999). Results of a molecular typing study of *C. jejuni* isolates from farm animals by Fitzgerald and colleagues demonstrated a link between *Campylobacter* isolates from farm animals with isolates from human cases within the community (Fitzgerald et al., 2001). Other studies have shown a correlation between *C. jejuni* infections and people exposed to farm animals (Kapperud et al., 1992; Friedman et al., 2004). A recent detailed study of *Campylobacter* in cattle revealed *Campylobacter* prevalences of approximately 59% in one large Michigan dairy herd and 62% and 84% in two beef herds (Cha et al., 2017).

Many studies over the past decade have examined *C. jejuni* populations in agricultural cattle in many countries worldwide. Most of these studies have been focused on determining prevalence and antimicrobial resistance patterns, with molecular verification methods usually including PCR confirmation of selected virulence-associated genes. Fewer authors have reported detailed multilocus sequence typing of collections of *C. jejuni* strains isolated from dairy or beef cattle and comparison of the types discovered to types found in human clinical isolates from the same geographic region. Molecular typing methods have included multilocus sequence typing (MLST), pulsed field electrophoresis (PFGE), *fla*A or *fla*B restriction fragment polymorphism (RFLP) analysis, comparative genomic fingerprinting (CGF), and whole genomic sequencing (WGS); many papers report results from more than one method, and many studies included antibiotic resistance profiling by either molecular or phenotypic methods. Only a few studies have included or been focused specifically on calves.

These studies have been performed in Africa [Nigeria (Ngulukun et al., 2016), Ethiopia (Nigatu et al., 2015; Hagos et al., 2021), South Africa (Karama et al., 2020)], Eastern Asia [China (Ju et al., 2018; Zang et al., 2021a), Japan (Ono, 2013; Mori et al., 2015; Kiatsomphob et al., 2019), Malaysia (Premarathne et al., 2017), South Korea (Dong et al., 2016; An et al., 2018)], Western Asia [Iraq (Khalifa et al., 2013a,b; Al-Edany et al., 2015) and India (Rajagunalan et al., 2014)], Europe [Austria (Klein-Joebstl et al., 2016), France (Thepault et al., 2018), Italy (Bianchini et al., 2014a,b), Latvia (Meistere et al., 2019; Terentjeva et al., 2019), Lithuania (Ramonaite et al., 2017; Aksomaitiene et al., 2019), Poland (Wieczorek and Osek, 2018), Spain (Ocejo et al., 2019), Sweden (Hansson et al., 2021), New Zealand (Rapp et al., 2012; Irshad et al., 2016)], and North America [Canada (Webb et al., 2018; Farfan et al., 2019; Inglis et al., 2020), and the United States (Cha et al., 2016; Tang et al., 2017)].

These studies can be broadly summarized as follows. Although the arrays of molecular sequence types varied between locations, not surprisingly, most authors found similar *C. jejuni* sequence types circulating both in cattle populations and among human clinical isolates in the geographic region studied. Antibiotic resistance was widespread in most studies in which it was examined, with many isolates carrying resistance to multiple antibiotics. Overall, considerable variation has been found in the number of positive animals, the amount of *Campylobacter* shed per positive animal, the constancy or intermittency of shedding, and the seasonality of shedding. Results were similar in those few studies focused specifically on calves or on a mixture of young and adult animals in which calves and adults were reported separately rather than studies focused on only adult cattle or on undifferentiated mixtures of ages (Klein-Joebstl et al., 2016; Meistere et al., 2019; Terentjeva et al., 2019; Hansson et al., 2021). The most relevant studies for comparison to the isolates reported here are those of Cha et al. (2016, 2017) and Rodrigues et al. (2021) in Michigan, United States. One dairy and two beef herds were studied; 22 *C. jejuni* STs were detected among 148 isolates from cattle; eight STs in this collection were also detected in a contemporaneous collection of 94 human clinical isolates from the Michigan Department of Health and Human Services (Cha et al., 2016), including four of the five sequence types recovered from calves in the small study reported here. Cha et al. (2017) concluded that cattle could be an important reservoir of *C. jejuni* in Michigan.

Guillain Barré Syndrome (GBS) is an acute neuromuscular autoimmune disease of the peripheral nervous system and is the most common cause of acute flaccid paralysis since the near eradication of polio (Church Potter and Kaneene, 2003; Poropatich et al., 2010). Symptoms may start with limb weakness and loss of tendon reflexes, but may develop into paralysis of the limbs, trunk, and facial muscles; 25% of patients may require mechanical ventilation (Hughes and Rees, 1994; Jacobs et al., 2008; Walling and Dickson, 2013). Ten to twenty percent of patients do not completely recover and have life-long disabilities; 3–10% die (Ang et al., 2004; Walling and Dickson, 2013). It has been estimated that the annual incidence of GBS is 1–2 people per 100,000 people in North America and Europe (Shui et al., 2012).

Guillain Barré Syndrome commonly develops following gastrointestinal infection with *C. jejuni*; one systematic review of published studies determined that *C. jejuni* infection preceded 13–72% of GBS cases (Poropatich et al., 2010). Molecular mimicry of lipo-oligosaccharide (LOS) structures found on the outer membrane of *C. jejuni* resembling gangliosides enriched in nervous tissue is thought to be the mechanism behind the pathogenesis of GBS (Ang et al., 2004; Szymanski and Gaynor, 2012). In particular, development of GBS has been associated with sialylation of LOS by the CMP-*N*-acetylneuraminate-beta-galactosamide-alpha-2,3-sialyltransferase (EC 2.4.99.-) and CMP-*N*-acetylneuraminate-beta-galactosamide-alpha-2,3, alpha 2,8-sialyltransferase (EC 2.4.99.-) enzymes encoded by *cst*II and *cst*III (Godschalk et al., 2007; Yuki, 2007). Enhanced risk of developing GBS has been recognized in agricultural workers who handle poultry and cattle. For example, Price et al. (2007) demonstrated an increase in development of peripheral neuropathy in people who work with poultry, and Vegosen et al. (2012) demonstrated that neurological symptoms associated with exposure to *C. jejuni* are more commonly reported among people who handle beef cattle. In addition, Vegosen et al. (2015) found that farm workers exposed to swine, which also often carry *C. jejuni*, had a trend toward increased levels of antibodies reactive with *C. jejuni* compared to non-farm workers. These farm workers did not exhibit increased levels of antibodies reactive with five different neurogangliosides; however, the LOS type(s) of *C. jejuni* circulating in the swine was(were) not known. These results indicate that it is possible for exposure to farm animals carrying *C. jejuni* with GBS-associated LOS types to trigger the formation of anti-ganglioside antibodies. In addition to LOS, some heat stable (HS) Penner serotypes of C. *jejuni*, derived from the capsule, have been associated with the development of GBS (Poly et al., 2011; Heikema et al., 2015; Poly et al., 2015; Liang et al., 2016). A survey by Zang et al. (2021a,b) found such serotypes prevalent in livestock in China, including in cattle.

In this study, residents of a family-owned southwest Michigan dairy farm complained of recurring *C. jejuni* infections over a course of 2 years. Stool samples were taken from 25 calves and 1 dog on the dairy farm and tested for presence of *C. jejuni*. Thirteen of twenty-five calves tested positive for *C. jejuni* (52%). We hypothesized that transmission of *C. jejuni* was occurring between the family members and the calves. To test this hypothesis, we used multiple molecular typing schemes to determine the genetic relationships among the *C. jejuni* isolates. Isolates were examined using multi-locus sequence typing (MLST), LOS locus class determination by PCR, *fla*A SVR typing, and *por*A typing.

## Materials and Methods

### Case Study

Residents of a family-owned mid-sized dairy farm in mid-Michigan reported *C. jejuni* infection among the family that lasted several days in summer, 2012. The family had experienced previous recurring *C. jejuni* infections for the previous 2 years. A veterinarian obtained 2 stool samples from dairy calves to eliminate them as possible carriers of *C. jejuni* and sent the samples to the Veterinary Diagnostic Laboratory (VDL) at Michigan State University (MSU) in East Lansing, MI, United States. Using bacteriological tests, VDL staff confirmed that the stool samples were positive for *C. jejuni*. VDL staff then contacted our laboratory, the Comparative Enteric Disease Laboratory at MSU. We contacted the veterinarian, who obtained 25 randomly chosen calf samples along with one stool sample from the family dog and sent them directly to our laboratory. While this was done following the calf sampling, we participated in a human study that was performed for people living in contact with cattle in Michigan. These are referenced in the paper.

### Ethics Statement

All protocols were approved by the Institutional Review Boards at MSU (IRB# 10-736SM) and the MDHHS (842-PHALAB).

### *Campylobacter* Isolation

Stool samples from the calves and dog were subcultured on Tryptone soy agar plates containing 5% sheep’s blood (Cleveland Scientific, Bath, OH, United States) and supplemented with antibiotics (TSA-CVA: 20 μg/mL cefoperazone, 10 μg/mL vancomycin and 2 μg/mL amphotericin B) upon arrival. All human clinical isolates were subcultured onto Bolton agar (Bolton Broth, Thermo Fisher Scientific, Pittsburgh, PA, United States) containing 1.5% Bacteriological Agar (Neogen, Lansing, MI, United States) upon arrival in the laboratory. All plates were placed in anaerobic jars containing CampyGen packs (Oxoid, Basingstoke, United Kingdom) and incubated for 48 h at 37°C. Isolates were selected and streaked again for growth in Tryptone Soy agar (TSA) and purification; bacterial growth was collected and stored at –80°C in TSB + 15% glycerol until further use.

### Preparation of Genomic DNA and Confirmation of *Campylobacter* spp.

Following growth on Bolton agar for 48 h at 37°C in anaerobic jars with CampyGen packs, DNA was extracted using Wizard^®^ Genomic DNA Purification Kits (Promega, Madison, WI, United States) according to the manufacturer’s instructions. All genomic DNA was diluted to a final concentration of 25 ng/μl for PCR.

Identification of *Campylobacter* isolates as *C. jejuni* or *C. coli* was performed with multiplex PCR as previously described (Linton et al., 1996). Hot Start Syzygy Mean Green DNA polymerase was used (Integrated Scientific Solutions, San Diego, CA, United States). The reaction had an enzyme activation step of 15 min at 95°C followed by 25 cycles of denaturation for 30 s at 95°C, annealing for 1.5 min at 58°C, and extension for 1 min at 72°C, with a final extension of 7 min at 72°C.

### Multi-Locus Sequence Typing, *por*A, and *fla*A Short Variable Regions Typing

Multi-locus sequence typing based on 7 conserved housekeeping genes (Dingle et al., 2001) was performed on DNA from the calf isolates as previously described (Bell et al., 2009, 2013); only the inner primers were used to amplify the partial gene sequences. Platinum^®^ Taq DNA Polymerase High Fidelity (Life Technologies, Grand Island, NY, United States) was used; primer concentrations used were 37.5 pmol. The PCR reaction contained 35 cycles of denaturation of 2 min at 95°C, annealing for 1 min at 50°C, followed by an extension for 1 min at 72°C. Human clinical isolates recovered by the MDHHS were previously characterized by MLST as described previously (Cha et al., 2016). Briefly, 10 μM of both the forward and reverse primer, 25 ng/μl of genomic DNA template, water, and the KAPA2G HotStart DNA polymerase (KAPABiosystems, Woburn, MA, United States) were used for the reaction. The reactions began with an enzyme activation step of 3 min at 95°C and consisted of 35 cycles of denaturation for 15 s at 95°C, annealing for 15 s at 60°C, and an extension for 5 s at 72°C.

*por*A allele typing was done using 7 different primers previously defined on the PubMLST website^1^ (Jolley and Maiden, 2010). The PCR reaction consisted of 40 cycles of denaturation for 30 s at 94°C followed by an annealing step for 30 s at 45°C and extension for 90 s at 72°C. Platinum^®^ Taq DNA Polymerase High Fidelity (Life Technologies, Grand Island, NY, United States) was used with 40 pmol of primer.

The *fla*A short variable regions (SVR) sequence typing was done with Platinum^®^ Taq DNA Polymerase High Fidelity (Life Technologies, Grand Island, NY, United States) and 40 pmol of primer. The PCR reaction consisted of a denaturation step at 94°C for 1 min, annealing at 45°C for 1 min, and extension for 3 min at 72°C for 35 cycles. The primers used were previously described (Meinersmann et al., 1997).

PCR products were purified with QIAquick PCR Purification Kit (Qiagen, Germantown, MD, United States) according to the manufacturer’s instructions and submitted for Sanger sequencing to the MSU Genomics Technology Support Facility (East Lansing, MI, United States) on an ABI 3730 Genetic Analyzer (Life Technologies, Grand Island, NY, United States). Each PCR product was sequenced in both the forward and reverse directions. SeqMan 5.06 (DNASTAR, Madison, WI, United States) software was used to align the sequences; the PubMLST website was used to determine allele, sequence type, and clonal complex assignments (Jolley and Maiden, 2010)^2^.

### Classification of Lipooligosaccharide Biosynthesis Loci

Lipooligosaccharide typing was performed by long-range PCR and restriction fragment polymorphism (RFLP) analysis as previously described by Parker et al. (2005) using DNA samples extracted as described above. This method can differentiate LOS classes A through F. Parker et al. (2005, 2008) described 19 classes (A–S) of LOS based on gene content subsequently, Richards et al. (2013) described an additional four classes (T-W). LOS locus class typing was confirmed using sequence data from gene content analysis of whole genome sequences as described below.

### Whole-Genome Sequencing and Analysis

DNA samples extracted as described above were submitted to the MSU RTSF for library construction and sequencing. Briefly, bacterial genomic DNA was prepared for sequencing using the TruSeq DNA Nano Library Preparation Kit (Illumina, San Diego, CA, United States). Libraries were subjected to quality control and quantitated using a Qubit dsDNA assay, Caliper LabChipGX and Kapa Library Quantitation qPCR Kit. Libraries were pooled in equimolar amounts and loaded on an Illumina MiSeq standard flow cell (v2). Sequencing was performed in a 2 × 250 bp paired end format with a 500-cycle reagent cartridge (v2). Base calling was done by Illumina Real Time Analysis (RTA) v1.18.54 and output of RTA was demultiplexed and converted to FastQ format with Illumina Bcl2fastq v1.8.4. The total number of reads was 16,751,374 and overall% ≥ Q30 was 88.4; sequence yield per genome is given in Table 1.

**TABLE 1:** Strains used in this study.

Strain	PATRIC/RAST accession number	Closest neighbor in PATRIC/RAST	Sequence yield (Gbp)	Number of contigs	Genome size	Source
*Campylobacter jejuni* LM01	6666666.157468	*C. jejuni* subsp. *jejuni* PT14	0.51	114	1871291	Calf; this study
*Campylobacter jejuni* LM03	6666666.157400	*C. jejuni* subsp. *jejuni* NCTC 11168	0.21	31	1695957	Calf; this study
*Campylobacter jejuni* LM05	6666666.157401	*C. jejuni* subsp. *jejuni* NCTC 11168	0.50	240	1913418	Calf; this study
*Campylobacter jejuni* LM08	6666666.157402	*C. jejuni* subsp. *jejuni* xy259	0.46	30	1787224	Calf; this study
*Campylobacter jejuni* LM10	6666666.157403	*C. jejuni* subsp. *jejuni* xy259	0.41	41	1783747	Calf; this study
*Campylobacter jejuni* LM11	6666666.157404	*C. jejuni* subsp. *jejuni* NCTC 11168	0.47	47	1792862	Calf; this study
*Campylobacter jejuni* LM12	6666666.157405	*C. jejuni* subsp. *jejuni* NCTC 11168	0.48	49	1711646	Calf; this study
*Campylobacter jejuni* LM13	6666666.157406	*C. jejuni* subsp. *jejuni* xy259	0.62	133	1824039	Calf; this study
*Campylobacter* spp. LM16^1^	6666666.157409	*C. fetus* subsp. *fetus* 82-40	0.51	522	2032764	Calf; this study
*Campylobacter jejuni* LM19	6666666.157411	*C. jejuni* subsp. *jejuni* PT14	0.66	71	1827399	Calf; this study
*Campylobacter* spp. LM20^1^	6666666.157413	*C. fetus* subsp. *fetus* 82-40	0.61	1054	2501437	Calf; this study
*Campylobacter jejuni* LM21	6666666.157414	*C. jejuni* subsp. *jejuni* PT14	0.61	107	1849874	Calf; this study
*Campylobacter jejuni* LM24	6666666.157415	*C. jejuni* subsp. *jejuni* xy259	0.60	93	1739756	Calf; this study
*Campylobacter jejuni* LM26	6666666.157416	*C. jejuni* subsp. *jejuni* xy259	0.48	126	1773940	Calf; MSU VDL^2^
*Campylobacter jejuni* LM27	6666666.157417	*C. jejuni* subsp. *jejuni* xy259	0.52	864	1597285	Calf; MSU VDL^2^
*Campylobacter jejuni* TW16491	6666666.157418	*C. jejuni* subsp. *jejuni* NCTC 11168	0.63	81	1725639	Human; MDCH^3^

*^1^Identified as C. hyointestinalis by ribosomal protein MLST with 95% support (PubMLST, https://pubmlst.org/species-id).*

*^2^Michigan State University Veterinary Diagnostic Laboratory.*

*^3^State of Michigan Department of Community Health/Thomas Whittam/Shannon Manning.*

FastQ files were uploaded to the Pathosystems Resource Integration Center (PATRIC) system (Brettin et al., 2015; Wattam et al., 2018) on 11/15/2015, for quality checking and assembly followed by annotation, and analysis in Classic RAST. Accession numbers are given in Table 1.

Lipooligosaccharide biosynthesis loci were examined in the genome sequences by performing protein homology BLAST searches. Presence or absence of all 54 LOS open reading frames (orfs) described previously (Parker et al., 2005, 2008; Richards et al., 2013) was determined using the BLAST utility in RAST https://rast.nmpdr.org/ (Aziz et al., 2008; Overbeek et al., 2014; Brettin et al., 2015; Wattam et al., 2018). Accession numbers for query sequences and detailed results are given in Supplementary Table 1; *E* values ≤ 1 × 10^––30^ were taken as positive hits for this analysis. Content of the LOS complex loci in each isolate was determined by the presence or absence of contiguous open reading frames giving positive hits that contained homologs of orf1 [UDP-glucose 4-epimerase (EC 5.1.3.2)], orf 2 [Lipid A biosynthesis lauroyl acyltransferase (EC 2.3.1.-)], orf3 [Lipopolysaccharide heptosyltransferase I (EC 2.4.1.-)] and orfs 12 (beta-1,3-galactosyltransferase/beta-1,4-galactosyltransferase) and 13 [D-glycero-D-manno-heptose 1,7-bisphosphate phosphatase (EC 3.1.1.-)]. Multiple alignment of *cst*II and *cst*III homologs was conducted using Clustal Omega^3^ (Sievers et al., 2011).

Presence and absence of LOS open reading frames and genomic complements of 1270 open reading frames with functions unambiguously identified by RAST were compared to the composition of the LOS classes defined in Parker et al. (2005, 2008) and Richards et al. (2013) using the Sorensen binary similarity coefficient and hierarchical clustering with unweighted pair groups and arithmetic averages (UPGMA) in PAST 2.12 software (Hammer et al., 2001). Multiple alignment of *cst*II and *cst*III homologs was conducted using Clustal Omega (see footnote 4; Sievers et al., 2011). Sequences for known *cst*II and *cst*III loci were obtained from GenBank (*cst*II, AF215639; strain ATCC43432, Houliston et al., 2006); *cst*III, AF257460, strain MSC57360 (Guerry et al., 2000) and genome sequences AL111168.1 (strain 11168) and AANK00000000 (strain 260.94).

Sequence-based comparisons to the following *C. coli* and clinical *C. jejuni* isolates were made using tools in the RAST SEED database: *C. coli* RM2228, *C. jejuni* subsp. *doylei* 267.97, *C. jejuni* subsp. *jejuni* RM1221 (chicken), and *C. jejuni* subsp. *jejuni* clinical isolates 11168 and 81–176 (enteritis); 84–25 (meningitis); 260.94 and HB93-13 (Guillain Barré Syndrome); and CF93-13 (Miller Fisher Syndrome). RAST accession numbers are 306254, 360109, 195099, 192222, 354242.8, 360110.3, 360108.3, 360112.3, and 360111.3, respectively.

Virulence-associated gene content (virulome) was assessed as follows. Promoter (TTCTTTAAATTTTATGATTT TACAATGAAATTAGTTATAATTGTAGTTAGGAT) and termination sequences (TAGTAATGATAGTAATGA) were added to back-translated nucleotide sequences for virulence-associated loci in *C. jejuni* 11168 and other strains and the resulting sequences concatenated to form an artificial chromosome, which was uploaded into PATRIC (accession number 186572). Accession numbers of the sequences used are given in Supplementary Table 3; promoter and terminator sequences were developed based on prior reports (Wosten et al., 1998; Lodish et al., 2000). Protein identity comparisons of this artificial chromosome were made to all isolates using RAST; *C. jejuni* 11168 (RAST accession 192222.1) was included in each comparison for quality control checking.

Full data for (1) LOS locus gene content of calf and human isolates reported here, (2) whole genome content of calf and human isolates reported here plus *C. jejuni* clinical strains 11168, 260.94, HB93-13, CF93-6, 84-25, and 81-176, *C. jejuni* chicken isolate RM1221, *C. jejuni* subsp. *doylei* 269.97, and *C. coli* RM2228; and (3) virulence-associated gene content of calf and human isolates reported here plus *C. jejuni* clinical strains 11168, 260.94, HB93-13, CF93-6, 84-25, and 81-176 are provided in Supplementary Tables 1–3, respectively.

### Gentamicin Killing Assay

1.5 × 10^6^ Caco-2 cells were grown in Dulbecco’s modified Eagle’s (DMEM) medium with 10% FBS and 2 mM L-glutamine at 37°C and 5% CO_2_. At confluency, cells were dissociated from the flask using 3 ml trypsin-EDTA (0.25%), resuspended in the same medium, washed, counted, and plated in 20 well plates at approximately 5 × 10^4^ cells per well (in 1 ml medium). Cells were incubated for 48 h at 37°C in 5% CO_2_ and used when confluency was ascertained. Three replicate wells of each experimental *C. jejuni* calf strain and the asymptomatic human strain (TW16491) strain were run with a negative control sham-inoculated with medium with no *C. jejuni* and three positive control wells with *C. jejuni* 11168 that is highly invasive. *C. jejuni* strains were then added at a multiplicity of infection of 100 followed by a 4-h incubation. For measuring invasion, cells were further incubated for 1 h with 250 μg/ml gentamicin, washed in PBS, lysed in 0.1% Triton X-100 and the released bacteria enumerated by serial dilution onto the surface of Bolton agar plates. Dilutions of 10^–1^, 10^–2^, 10^–3^, and 10^–4^ were streaked onto fresh Bolton agar plates and incubated for 48 h in 5% CO_2_. Then plates were removed from the incubator and colonies counted. On the day of infection of the Caco-2 cells the inocula for each *C. jejuni* strain were diluted in 10-fold dilutions and assayed by the same limiting dilution method to determine the actual number of cfu in the inoculum for each strain.

We also conducted a broth microdilution assay to determine the concentration of antibiotic necessary to kill the extracellular *C. jejuni* of all strains (11168, TW16491, LM01, LM08, LM11, LM12, LM24) in the gentamicin killing assay. Briefly, *C. jejuni* strains were grown for 48 h on Bolton agar plates in 5% CO2 at 37°C. Plates were swabbed and each strain suspended in 2 ml Bolton broth, the concentration determined at OD600 and the concentration adjusted to ∼1.0 at OD100. Thereafter, we diluted each strain suspension 1:10 in Bolton broth and added 10ul cell suspensions to each well of a 96 well plate according to the plate map. Based on previous titrations, about 1 × 10^6^ cells were added per well. Then, Gentamicin was added to the 12 wells of each *C. jejuni* strain in decreasing concentrations from 400, 200, 100, 50, 25, 12.5, 8.375, 4.1875, 2.09, 1.04, 0 μg/ml. The plate was incubated for 20 h, in 5% CO_2_ at 37°C. Results of the broth microdilution method are reported in minimum inhibitory concentration (MIC), or the lowest concentration of antibiotics that stopped bacterial growth.

## Results

### Verification of *Campylobacter jejuni* and Clinical Illness

Seventeen of twenty-five calf stool samples were positive for *Campylobacter* spp.*;* the dog sample was negative. Two isolates recovered from the calves were identified as *C. coli* but were not examined further. The referring veterinarian who submitted the initial diagnostic calf samples reported that calves were in a sick pen because of intermittent diarrhea and no respiratory disease was evident. Calves were negative for viral and parasitic causes of diarrhea. Phone interviews revealed that it was a common practice during the 2-year period of diarrheal problems in the family to isolate calves with diarrhea in a pen in a barn near the house and that family members had cared for these calves over this time. Adult cattle on the farm were managed in a facility more distant from the calves by caretakers hired for this purpose. The referring veterinarian had ruled out the cows as the source of the family’s gastroenteritis, so no further diagnostics were done on cows.

Family members, three children and two parents, had no symptoms of *C. jejuni* infection at the time of stool sampling. Of the five family members, one of the children tested positive for *C. jejuni*; therefore, this child was considered an asymptomatic carrier. In previous studies, two of these family members with campylobacteriosis presented to a local hospital where *C. jejuni* isolates were obtained by MDHHS and characterized by the Manning laboratory (Cha et al., 2016; Rodrigues et al., 2021). Epidemiologic descriptions of these infections have been published (Cha et al., 2016; Rodrigues et al., 2021). In the first case, contact with cattle, dogs and cats was reported (Cha et al., 2016), while in the second case living in urban areas and traveling including international travel were reported (Rodrigues et al., 2021).

Fifteen calf isolates were tentatively identified as *C. jejuni* by multiplex PCR typing (Table 1). Genome sequences were obtained for these fifteen isolates as well as the human isolate. Species identifications were also confirmed by comparing ribosomal protein sequences to the PubMLST database^4^ (Jolley and Maiden, 2010; Jolley et al., 2012); two isolates (LM16 and LM20) were more likely to be *C. hyointestinalis* than *C. jejuni* (Table 1). Comparison of the LM16 16S rDNA sequence to all *Campylobacter* isolates in GenBank (Accessed March 2021) also indicated that the isolate was likely *C. hyointestinalis*. The more fragmentary nature of the genome sequences of *C. hyointestinalis* isolates LM16 and LM20 and *C. jejuni* isolate LM27 precluded extended analysis of those isolates.

### Multilocus Sequence Typing Analysis, *por*A Allele Typing and *fla*A Short Variable Regions Sequence Typing, and Antibiotic Resistance

Multilocus sequence typing allelic profiles, *fla*A SVR, *por*A, and LOS typing results are shown in Table 2. Among all 13 isolates evaluated, five multilocus sequence types (STs) were identified that belonged to STs 806, 922, 929, 982, and 6227. ST assignments were confirmed using sequences extracted from the whole-genome sequences as well. The human isolate belonged to ST922 as did two other calf isolates. Members of each ST represented by multiple isolates had identical *fla*A and *por*A alleles with the exception of ST929; the three isolates of that ST all possessed different *fla*A alleles. Antibiotic resistance gene profiles extracted from the whole-genome sequences varied across the isolates and were consistent within STs except for isolate LM13. This isolate lacked putative aminoglycoside and tetracycline resistance genes found in the other three ST806 isolates (Table 3). The human isolate, TW16491, was previously found to be resistant to tetracycline, though aminoglycoside and β-lactam resistance was not evaluated (Cha et al., 2017).

**TABLE 2:** Molecular typing of *C. jejuni* isolates.

	MLST alleles*											
Strain	*asp*A	*gln*A	*glt*A	*gly*A	*pgm*	*tkt*	*unc*A	Sequence type (clonal complex)	*fla*A*	*por*A*	LOS type**	Penner serotype associated with GBS***
*Campylobacter jejuni* LM01	9	2	4	62	4	5	17	929 (257)	94	749	C	None
*Campylobacter jejuni* LM19	9	2	4	62	4	5	17	929 (257)	51	749	F	None
*Campylobacter jejuni* LM21	9	2	4	62	4	5	17	929 (257)	61	749	F	None
*Campylobacter jejuni* LM03	1	1	2	83	2	3	6	922 (NA)	81	2264	E	None
*Campylobacter jejuni* LM12	1	1	2	83	2	3	6	922 (NA)	81	2264	E	None
*Campylobacter jejuni* TW16491	1	1	2	83	2	3	6	922 (NA)	81	2264	E	None
*Campylobacter jejuni* LM08	2	1	1	3	140	3	5	806 (21)	95	749	B2	HS4A/B
*Campylobacter jejuni* LM10	2	1	1	3	140	3	5	806 (21)	95	749	B2	HS4A/B
*Campylobacter jejuni* LM13	2	1	1	3	140	3	5	806 (21)	95	749	B2	HS4A/B
*Campylobacter jejuni* LM26	2	1	1	3	140	3	5	806 (21)	65	749	B2	HS4A/B
*Campylobacter jejuni* LM05	2	1	1	362	2	1	6	6227 (21)	95	351	C	HS2
*Campylobacter jejuni* LM11	2	1	1	362	2	1	6	6227 (21)	95	351	A2	HS2
*Campylobacter jejuni* LM24	2	1	2	3	2	1	5	982 (21)	97	749	C	HS2

*Determined using whole genome sequence results.

**Determined by long range PCR/RFLP as described by Parker et al. (2005).

***Determined by BLAST as described in the text.

**TABLE 3:** Presence of ORFS encoding antibiotic resistance.

		Aminoglycoside modification			Beta lactamases		Tetracycline*
Strain	ST	Aminoglycoside acetyltransferase (EC 2.3.1.81)	Aminoglycoside phosphotransferase	Aminoglycoside 6-adenyltransferase	Beta-lactamase (EC 3.5.2.6)	Putative lactamase B	*tet*O
LM03	922	+	–	+	+	+	+
LM12	922	+	–	+	+	+	+
TW16491	922	+	–	+	+	+	+
LM01	929	+	+	–	+	+	+
LM19	929	+	+	–	+	+	+
LM21	929	+	+	–	+	+	+
LM08	806	+	+	–	+	+	+
LM10	806	+	+	–	+	+	+
LM13	806	+	–	–	+	+	–
LM26	806	+	+	–	+	+	+
LM05	6227	+	–	–	+	+	+
LM11	6227	+	–	–	+	+	+
LM24	982	+	+	–	+	+	+

*LM13 contains an ORF annotated as a tetracycline resistance-conferring translation elongation factor.

Clonal complex ST-21 accounted for half of the isolates in our study (STs 806, 982, and 6227).

### Lipooligosaccharide Classification

PCR/RFLP typing of twelve different genes in LOS biosynthesis loci assigned the four ST806 isolates to class B2 and the three ST922 isolates to class E, including the family member isolate. One ST6227 isolate, one ST929 isolate, and the single ST982 isolate were assigned to class C. Another ST6227 isolate was assigned to A2. The remaining two ST929 isolates were assigned to class F. Calf isolates LM03 and LM12 were found to have the same LOS class (E), MLST sequence type (ST922).

**FIGURE 1:**
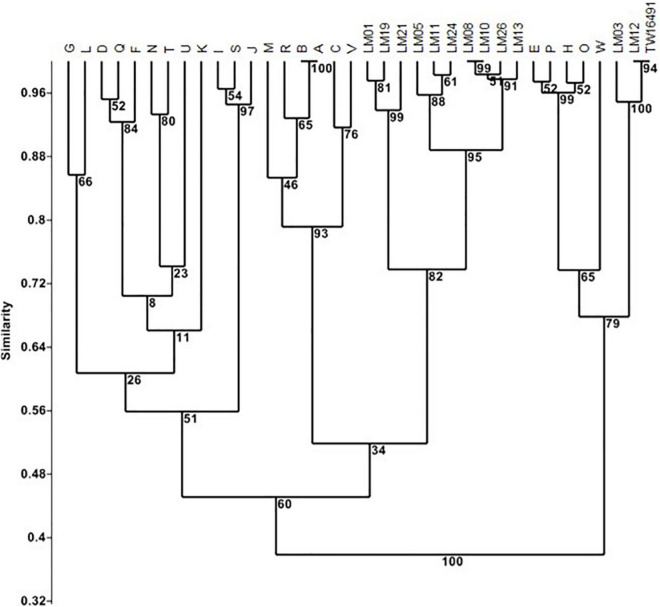
Cluster analysis of LOS locus content in calf and human isolates. Clustering was performed In PAST 2.12 (Hammer et al., 2001) using the Sorensen coefficient and the unweighted pair groups with arithmetic averages method on presence/absence data for 55 LOS loci in LOS classes A through W as detailed by Parker et al. (2005, 2008) and Richards et al. (2013). Numbers at nodes indicate the percentage of 1000 bootstrap replicates that support that node. Full data appear in Supplementary Table 1.

An independent analysis was based on LOS biosynthesis locus gene content determined from whole genome sequencing (Figure 1 and Supplementary Table 1) by conducting TBLASTN searches for each of the 55 loci described by Parker et al. (2005, 2008) and Richards et al. (2013); *E*-values of 1 × 10^–30^ were considered positive hits. The complex LOS loci were defined as contiguous open reading frames containing homologs to orfs 1, 2, 12, and 13 as described by Parker et al. (2005). In the case of isolate LM26, homologous open reading frames (ORFs) were divided between two sets of contiguous loci. This analysis showed that calf isolates LM03 and LM12 and the human isolate TW16941 clustered together with LOS classes E, H, O, and P (79% of bootstrap replicates) and that the LOS locus gene contents of LM12 and TW16491 were identical. The remainder of the calf isolates clustered with LOS classes A, B, C, M, R, and V (60% of bootstrap replicates). Note that LOS classes A and B are differentiated by allelic sequence differences that are not accounted for in this presence/absence analysis (Parker et al., 2005).

Protein-to-protein identity data indicated that all calf isolates possessed homologs of the loci *neu*A, *neu*B, and *neu*C encoding neuraminic acid biosynthesis. LOS locus class E lacks the genes needed to synthesize sialic acid (*neu*A, *neu*B, *neu*C) and transfer it to the LOS outer core sugar resides (*cst*II/*cst*III) (Parker et al., 2008); genomic analysis showed that isolates LM03, LM12, and TW16491 did potentially possess homologs to those five loci; however, the percent protein identity to the reference *neu*A, *neu*B, and *neu*C sequences was substantially lower than in other isolates in our sample.

**FIGURE 2:**
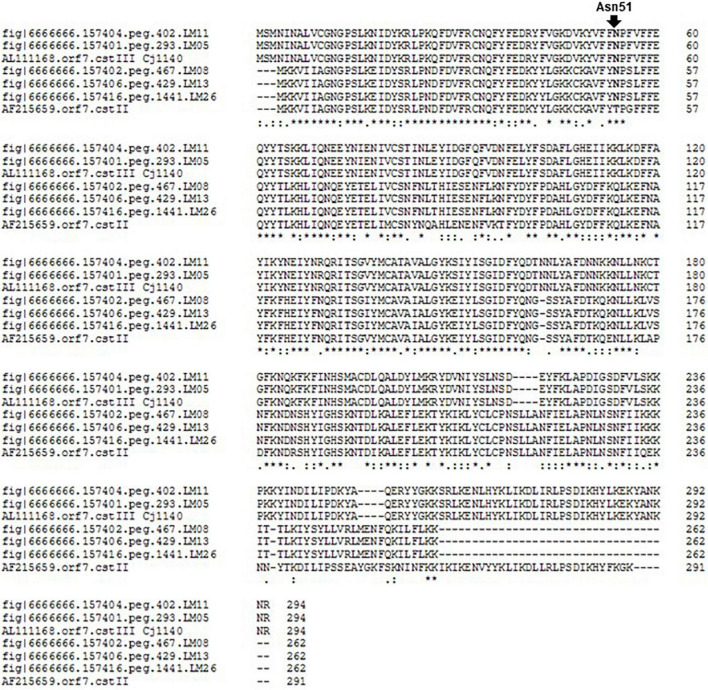
Alignment of translated calf isolate *cst*II/*cst*III homologs to known loci. Clustal Omega alignment of *cst*II/*cst*III homologs to known *cst*II and *cst*III sequences (AF215659, AF257460, Cj26094_1202, and AL111168 Cj1140, respectively). The position of residue Asn51 is indicated.

In addition, a number of calf isolates possessed homologs to the enzymes α-2,3-sialyltransferase (*cst*III) or α-2,3- and α-2,8- sialyltransferase (*cst*II); these enzymes attach neuraminic acid moieties to galactose residues in LOS to produce ganglioside mimics. Translation and alignment of the nucleotide sequences for those ORFs with known *cst*II and *cst*III protein sequences showed that calf isolates LM05, LM08, LM 11, LM13, LM24, and LM 26 had intact homologs of the reference loci and contained Asn51 residues (Figure 2). Thus, those isolates are potentially capable of producing α-2, 3-, α-2,8- di-sialylated LOS (Godschalk et al., 2007). We also note that while two ORFs homologous to *cst*II/*cst*III were detected in isolate LM01, the percent identities of the homologs in the ST922 isolates relative to the reference sequence were relatively low.

### Penner Serotypes

DNA sequence and primer information from Poly et al. (2011, 2015), was used to obtain the predicted PCR products expected for determination of each of eight heat stable (HS) Penner serotypes associated in the literature with GBS: HS1, HS2, HS4A, HS4B, HS19, HS23-36, HS41, and HS44 (Poly et al., 2011, 2015; Heikema et al., 2015; Liang et al., 2016). These predicted PCR products were used to probe the calf isolate genomes using BLAST. The two ST6227 strains (LOS types C and A2) and the ST982 strain (LOS type C) contained sequences with 100% homology to the probe for HS2, and the four ST806 (LOS type B2) strains contained sequences with 100% homology to the probes for both HS4A and HS4B. The ST929 and ST922 isolates reported here, including the human isolate TW16941, had no homology to any of the eight probes tested (Table 2).

### Whole Genome Level Comparisons

**FIGURE 3:**
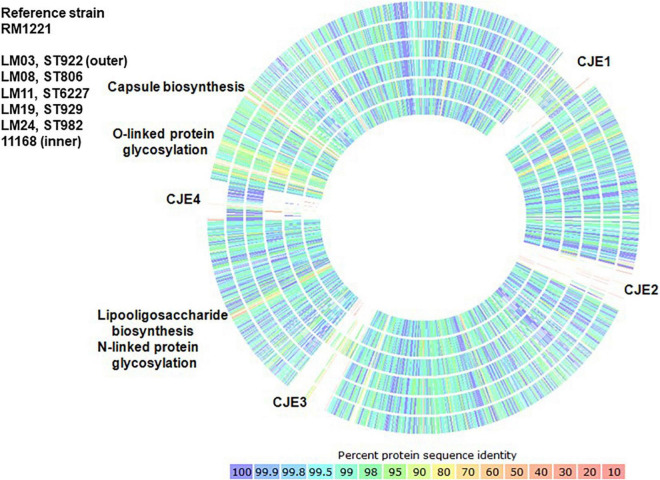
Comparison of percent protein identity of one representative strain of each ST to reference strain *C. jejuni* RM1221 as determined in RAST. Colors indicate protein percent identity of open reading frames within the strains of each sequence type in this study and *C. jejuni* 11168 (Parkhill et al., 2000) to reference strain *C. jejuni* RM1221 (Parker et al., 2006), which does not appear in the graph.

Genetic relationships among the thirteen isolates reported here, six clinical *C. jejuni* strains, (260.94, HB93-13, CF93-6, 84-25, 81-176, and 11168), *C. jejuni* chicken isolate RM1221, *C. jejuni* subsp. *doylei* 269.97, and *C. coli* RM2228 were visualized using percent identity protein-to-protein sequence comparisons in RAST (Figure 3 and Supplementary Figure 1A though F, and Supplementary Table 2). *C. jejuni* strain RM1221 was used as the reference strain for all visual comparisons in Figure 3 so as to illustrate the presence or absence of genomic islands CJE1 through CJE4 described by Parker et al. (2006); strain NCTC 11168, which does not possess any of the genomic islands, was included as a further reference. Strain RM1221 does not appear on the image; gaps in the comparison of RM1221 to strain NCTC 11168 (inner circle) indicate their positions in the genome. A single representative of each ST was included in Figure 3 to simplify comparison to *C. jejuni* 11168; comparison of one strain of each ST to five additional clinical strains (260.94, HB93-13, CF93-6, 84-25, and 81-176) is shown in the Supplementary Figure 1A. Isolates within each ST had similar patterns; comparisons of isolates of each ST to the six clinical strains (260.94, HB93-13, CF93-6, 84-25, 81-176, and 11168) shown individually in the Supplementary Figure 1B through F. Like *C. jejuni* 11168, all ST922 and ST929 isolates and the ST982 isolate largely lacked all four genomic islands; ST806 and ST6227 isolates lacked islands CJE2 and CJE3 but possessed islands CJE1 and CJE4.

**FIGURE 4:**
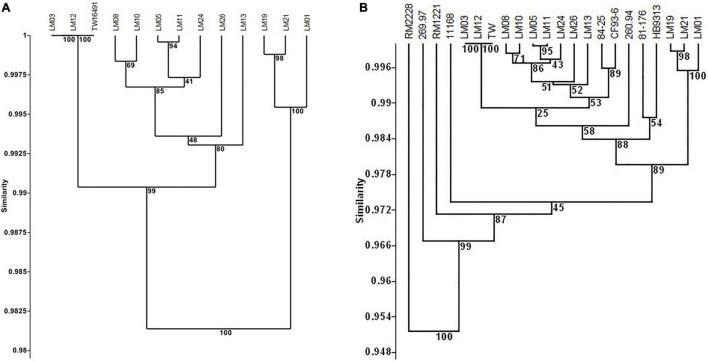
Cluster analysis of content of 1270 unambiguously identified open reading frames in calf and human isolates. UPGMA clustering was performed as in Figure 1 using the Sorensen coefficient (presence/absence of homologs) on data for 1270 open reading frames in reference strain LM01 that had unambiguously defined functions as determined in RAST using isolate LM01 as the reference strain. Numbers at nodes indicate the percentage of 1000 bootstrap replicates that supported that node. Detailed data used in the cluster analyses are given in Supplementary Table 4. **(A)** Twelve calf isolates and TW16941; **(B)** twelve calf isolates, TW16491, and six clinical isolates: 11168 and 81-176 (enteritis); 84-25 (meningitis); 260.94 and HB93-13 (Guillain Barré Syndrome); and CF93-13 (Miller Fisher Syndrome). Full data appear in Supplementary Table 2.

Because it had the largest genome among the 13 isolates whose genomes were examined, isolate LM01 was chosen as the reference strain for percent protein identity comparisons among the isolates described here. Results are given in Figures 4A,B and Supplementary Table 2. In summary, functions were unambiguously assigned to 1270 ORFs; 212 ORFs were designated as having probable, possible, or putative functions, and a further 424 were designated as hypothetical proteins. Seventy-four orfs were associated with plasmid or bacteriophage origins. When strain LM01 was used as the reference strain and presence or absence of the 1270 unambiguously identified ORFs scored, it appeared that, within the limits of short read sequencing technology, no two genomes had identical complements of ORFs (Figure 4A and Supplementary Table 2). It is also important to note that percent sequence identity comparisons should be interpreted with considerable caution, especially so in the case of the *C. jejuni* complex loci encoding biosynthesis of surface structures, which contain many intrachromosomal partial homologs (Parkhill et al., 2000; Parker et al., 2006). Nevertheless, this analysis again indicates the near identity of ST922 calf isolates LM03 and LM12 with the human isolate TW16491. Only eight of the 1270 ORFs having unambiguously identified functions differed among the three ST922 isolates (Table 4); the differences were minor except for aldehyde dehydrogenase A (EC 1.2.1.22)/glycolaldehyde dehydrogenase (EC 1.2.1.21) and 3-oxoacyl-[acyl-carrier-protein] synthase, KASIII (EC 2.3.1.41), TW16491 differed from LM03 or LM12 in those two ORFs. The divergence in the gene content of LM13 and LM26 from the other two ST806 isolates (LM08 and LM10) was unexpected.

**TABLE 4:** Virulence-associated loci variable between ST922 isolates.

Strain		LM03	LM12	TW16491

	RAST accession number	157400	157405	157418
		
Function	LM01 peg number	Percent identity to LM01 orf as determined in RAST		
Aldehyde dehydrogenase A (EC 1.2.1.22)/Glycolaldehyde dehydrogenase (EC 1.2.1.21)	fig| 6666666.157468.peg.731	28.89	28.89	100
3-oxoacyl-[acyl-carrier-protein] synthase, KASIII (EC 2.3.1.41)	fig| 6666666.157468.peg.845	31.55	31.55	97.96
Motility accessory factor	fig| 6666666.157468.peg.868	60.92	63.05	63.05
CMP-*N*-acetylneuraminate-beta-galactosamide-alpha-2,3-sialyltransferase (EC 2.4.99.-)	fig| 6666666.157468.peg.941	53.28	56.84	53.28
GDP-mannose 4,6-dehydratase (EC 4.2.1.47)	fig| 6666666.157468.peg.945	88.66	93.88	88.66
CMP-*N*-acetylneuraminate-beta-galactosamide-alpha-2,3-sialyltransferase (EC 2.4.99.-)	fig| 6666666.157468.peg.949	52	60.17	52
Haemin uptake system outer membrane receptor	fig| 6666666.157468.peg.1471	95.42	87.01	87.01
C4-dicarboxylate transporter	fig| 6666666.157468.peg.1716	100	100	98

The dendrogram in Figure 4B shows a similar analysis of the 1270 unambiguously identified ORFs that includes clinical *C. jejuni* isolates 11168, 260.94, HB93-13, CF93-6, 84-25, 81-176, and *C. jejuni* chicken isolate RM1221, *C. jejuni* subsp. *doylei* 269.97, and *C. coli* RM2228. Full data appear in Supplementary Table 2. ST922, ST929, and ST6227 isolates clustered together, while ST806 isolates LM13 and LM26 were again separated in gene content from LM08 and LM10. All 13 isolates reported here clustered with all six clinical strains (11168, 84–25, CF93-6, 260.94, HB93-13, and 81–176) and separately from outgroup strains chicken isolate *C. jejuni* RM1221, *C. jejuni* subsp. *doylei* 269.97, and *C. coli* RM2228 (87% bootstrap support).

**FIGURE 5:**
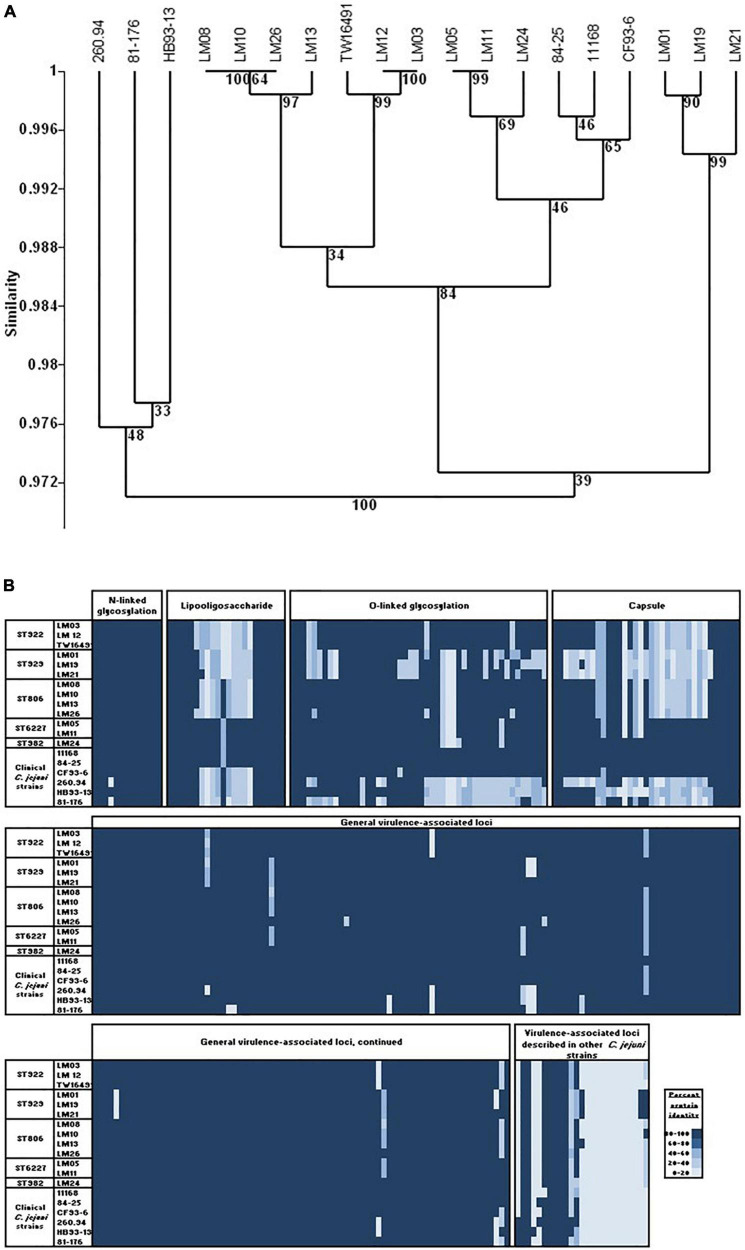
Virulence-associated ORFs in calf and human isolates. **(A)** UPGMA clustering was performed as in Figure 1 using the Sorensen coefficient (presence/absence of homologs) on data for 342 ORFs associated with virulence in *C. jejuni*. **(B)** Heat map showing protein% identity of open reading frames in calf isolates to the *C. jejuni* virulome; variation within and between sequence types. Full data appear in Supplementary Table 3.

More detailed protein-to-protein BLAST comparisons were made of the isolate genomes to an updated set of *C. jejuni* virulence*-*associated loci (virulome; Jacobs et al., 2008; Baek et al., 2011; Buelow et al., 2011; Nielsen et al., 2011; Theoret et al., 2011, 2012; Barrero-Tobon and Hendrixson, 2012; Boehm et al., 2012; Cullen et al., 2012, 2013; Frirdich et al., 2012; Lertpiriyapong et al., 2012; Neal-McKinney and Konkel, 2012; Bell et al., 2013; Rasmussen et al., 2013; Salamaszynska-Guz et al., 2013; Samuelson and Konkel, 2013; Samuelson et al., 2013). ORFs not detected in one or more of the 13 isolates reported here are shown in Table 5. Full comparisons are presented visually in the dendrogram in Figure 5A and the heat map in Figure 5B; underlying data are given in Supplementary Table 3. The dendrogram in Figure 5A shows clustering based on presence or absence of 342 virulence-associated ORFs and includes clinical *C. jejuni* isolates 11168, 260.94, HB93-13, CF93-6, 84–25, and 81–176. ST922, ST929, and ST6227 isolates clustered together; in this analysis, ST806 isolates LM13 and LM26 were similar in virulence-associated gene content to LM08 and LM10. All thirteen isolates reported here clustered with clinical strains 11168, 84-25, and CF93-6 but were separated from isolates 260.94, HB93-13, and 81-176 (100% bootstrap support).

**TABLE 5:** Protein percent ID between variable *C. jejuni* virulence-associated genes and open reading frames as determined in RAST.

		ST922			ST806				ST929			ST6227		ST982
	Annotation from RAST	LM03	LM12	TW 16491	LM08	LM10	LM13	LM26	LM01	LM19	LM21	LM05	LM11	LM24
**(A) Complex loci encoding surface antigens in ***C. jejuni* 11168****														
**(1) Lipo-oligosaccharide biosynthesis locus**														
CJ1137c.LOS	Capsular polysaccharide synthesis protein	44.41	44.41	44.41	**0**	**0**	**0**	**76.25**	0	0	0	100	100	100
CJ1139WLAN/CGTB	Beta-1,3-galactosyltransferase/Beta-1,4-galactosyltransferase	39.74	39.74	39.74	**59.87**	**59.87**	**59.87**	**0**	54.55	54.55	54.55	100	100	100
CJ1144/45	FIG00471437: hypothetical protein	0	0	0	**0**	**0**	**0**	**70.16**	61.33	61.33	61.33	99.62	99.62	99.62
CJ1148.WAAF	ADP-heptose–lipooligosaccharide heptosyltransferase II (EC 2.4.1.-)	93.42	93.42	93.42	**94.98**	**94.98**	**94.98**	**0**	92.65	92.65	92.65	100	100	100
**(2) O-linked glycosylation locus**														
CJ1322	FIG00471415: hypothetical protein	100	100	100	**0**	**0**	**0**	**99.07**	0	0	0	0	0	0
CJ1323	FIG00470597: hypothetical protein	98.44	98.44	98.44	**0**	**0**	**0**	**95.24**	0	0	0	0	0	0
CJ1325/6	FIG00469667: hypothetical protein	98.21	98.32	98.21	**97.77**	**97.77**	**98.32**	**0**	78.47	76.99	98.32	99.16	99.16	95.4
CJ1327.NEUB2	Legionaminic acid synthase (EC 2.5.1.56)	97.6	97.6	97.6	**97.31**	**97.31**	**97.31**	**0**	94.69	81.44	81.44	97.9	97.9	97.01
**(2) Capsular polysaccharide biosynthesis locus**														
CJ1415c.CYSC	Adenylylsulfate kinase (EC 2.7.1.25)	100	100	100	98.24	98.24	98.24	99.51	0	0	0	100	100	100
CJ1418c.CAPS	Phosphoenolpyruvate synthase/Pyruvate phosphate dikinase	99.23	99.23	99.23	99.36	99.36	99.36	94.92	**0**	**92.26**	**0**	99.87	99.87	99.74
CJ1420c.CAPS	Methyltransferase (EC 2.1.1.-), possibly involved in *O*-methyl phosphoramidate capsule modification	98.44	98.44	98.44	100	100	100	99.6	0	0	0	100	100	100
CJ1426c.CAPS	Methyltransferase, FkbM family protein	0	0	0	0	0	0	46.73	0	0	0	0	0	100
CJ1429c.CAPS	FIG00469885: hypothetical protein	0	0	0	**0**	**0**	**0**	**97.35**	0	0	0	0	0	100
CJ1430c.RFBC	dTDP-4-dehydrorhamnose 3,5-epimerase (EC 5.1.3.13)	82.39	82.39	82.39	**82.39**	**82.39**	**82.39**	**0**	73.33	73.33	73.33	82.39	82.39	100
CJ1433c.CAPS	Hypothetical protein Cj1433c	0	0	0	0	0	0	0	0	0	0	98.13	98.13	100
CJ1435c.CAPS	Phosphoserine phosphatase (EC 3.1.3.3)	26.36	26.36	26.36	26.36	26.36	26.36	50.41	0	0	0	100	100	100
CJ1437c.CAPS	Histidinol-phosphate aminotransferase (EC 2.6.1.9)	29.68	29.68	29.68	29.68	29.68	29.68	0	29.68	29.68	29.68	100	100	100
CJ1439c.GLF	UDP-galactopyranose mutase (EC 5.4.99.9)	0	0	0	0	0	0	26.36	0	0	0	100	100	100
CJ1441c.KFID	UDP-glucose 6-dehydrogenase (EC 1.1.1.22)	0	0	0	0	0	0	29.68	27.15	27.15	27.15	100	100	100
CJ1442c.CAPS	Predicted glycosyltransferase involved in capsule biosynthesis	94.05	94.05	94.05	93.68	93.68	93.68	41.18	0	0	0	100	100	100
CJ1443c.KPSF	Capsular polysaccharide export system protein KpsF	96.83	96.83	96.83	**96.19**	**96.19**	**96.19**	**0**	95.56	95.56	95.56	100	100	100
CJ1445c.KPSE	Capsular polysaccharide export system inner membrane protein KpsE	97.85	97.85	97.85	**98.12**	**98.12**	**98.12**	**0**	97.58	97.58	97.58	100	100	100
CJD26997_1801	capsular polysaccharide biosynthesis protein	0	0	0	**0**	**0**	**0**	**100**	0	0	0	0	0	0
**(B) Other virulence-associated loci**														
CJ0628/9c.APA	Possible lipoprotein	89.21	89.21	89.21	88	88	88	88	**0**	**0**	**90.96**	88.88	88.88	88
CJ1555c	Rrf2-linked NADH-flavin reductase	0	0	0	100	100	100	100	100	100	100	100	100	100
CJ1677 + 1678c.APB	Possible lipoprotein	100	100	100	100	100	100	98.55	**0**	**0**	**90.96**	100	100	100
CJ81176_1344PGP1	FIG00638667: hypothetical protein	98.49	98.49	98.49	98.28	98.28	98.28	89.45	98.71	98.71	98.71	98.71	98.71	98.28
CJJ26094_0063RLOE	Translation-disabling ACNase RloC	0	0	0	**0**	**0**	**0**	**100**	0	0	0	0	0	0
CJJ81176_1647.FEDD	FIG00469787: hypothetical protein	96.67	96.67	96.67	**96.67**	**96.67**	**96.67**	**0**	96.67	96.67	96.67	96.67	96.67	96.67
CJJHB9313_0989.P95	Filamentous hemagglutinin domain protein	51.27	51.27	51.27	**65.17**	**65.17**	**65.17**	**0**	30.33	30.33	30.33	65.17	65.17	65.17
Cj108tagH	FIG00710473: hypothetical protein	0	0	0	**0**	**0**	**97.14**	**98.52**	98.66	98.66	98.66	0	0	0
Cj108tssM	IcmF-related protein	0	0	0	**0**	**0**	**0**	**99.05**	0	0	0	0	0	0
Cj108tssD	hcp protein	0	0	0	**0**	**0**	**0**	**99.25**	0	0	0	0	0	0
Cj108tssL	Outer membrane protein ImpK/VasF, OmpA/MotB domain	0	0	0	**0**	**0**	**0**	**100**	0	0	0	0	0	0
Cj108tssK	Uncharacterized protein ImpJ/VasE	0	0	0	0	0	0	41.41	0	0	0	0	0	0
Cj108tssJ	Type VI secretion lipoprotein/VasD	0	0	0	**0**	**0**	**0**	**95.77**	0	0	0	0	0	0
Cj108tssA	Uncharacterized protein ImpA	0	0	0	**0**	**0**	**0**	**100**	0	0	0	0	0	0
Cj108tssI	Type VI secretion protein/vgrG	0	0	0	0	0	0	0	**100**	**77.09**	**65.51**	0	0	0

*Bold values indicate loci differing among strains of a single MSLT sequence type.*

Data in Table 5 show that the presence or absence of most virulence associated ORFs was consistent within an ST. Differences within STs are shown in bold type. However, isolate LM26 possessed multiple ORFs that were not detected in the remaining three ST806 isolates but were found in isolates representing other STs in the sample set. In addition, isolate LM21 possessed homologs to Cj1325/1326 and Cj1677/1678 and isolate LM19 possessed a homolog to Cj1418 that were not detected in other ST929 isolates but were found in isolates belonging to other STs. These observations suggest the possibility of horizontal gene transfer at some point in the history of this population.

Presence/absence variation was most prevalent in loci associated with surface structures: four LOS biosynthesis loci, five O-linked glycosylation loci, and fourteen capsular biosynthesis loci. Loci associated with LOS and capsule biosynthesis and O-linked glycosylation comprise 31% of the 342 virulence-associated loci in the *C. jejuni* virulome but 58% of loci (showing presence/absence variation) of the homologous ORFs in the isolates studied here (Figure 5B and Supplementary Table 3). Only one non-surface structure encoding a virulence associated ORF, Cj1055c (phosphoglycerol transferase), that was absent in any of the calf isolates (ST929 strains), was not also absent in one or more clinical strains.

### All Calf Isolates Were Capable of Invading a Caco-2 Cell Model of the Intestinal Epithelium

We hypothesized that all *C. jejuni* isolated from the calves were capable of infecting humans. To test this, a gentamicin killing assay was conducted to determine the invasive potential of the calf-derived *C. jejuni* isolates in comparison to the asymptomatic human isolate and *C. jejuni* strain 11168 known to be associated with gastroenteritis. Five calf *C. jejuni* isolates representing different ST groups (LM01-ST929, LM08-ST806, LM11-ST6227, LM12-ST922, LM24-ST982) were compared to human *C. jejuni* isolates TW16491 (asymptomatic family member-derived in this study) and 11168 (from a patient with gastroenteritis) for their ability to invade Caco-2 cells using a gentamicin killing assay. All calf-derived *C. jejuni* strains cultured for the inocula grew readily in Bolton broth and displayed similar cfus and darting motility in mid-log phase cultures. When inoculated onto Caco-2 cells at a multiplicity of infection of 100 cfu/cell, all strains were capable of invading cells (Table 6). Interestingly, calf-derived isolates LM01, LM08, LM11, and LM12 invaded at higher rates than the positive invasive control C. *jejuni* 11168. Furthermore, calf-derived strain LM24 invaded at the same efficiency as the asymptomatic family member-derived *C. jejuni* TW16491 at a rate one log lower than *C. jejuni* 11168 and all other calf-derived strains. Regardless, this assay demonstrated that all of the calf-derived strains were capable of invading human colonic epithelial cells. Moreover, calf-derived LM12 had higher invasive potential *in vitro* than the asymptomatic human-derived strain TW16941, which both had classes E, H, O, and P LOS locus gene content. Finally, highly invasive calf-derived LM01, LM08, and LM11 isolates clustered with LOS classes A, B, C, M, R, and V, which are LOS classes capable of producing the molecular mimicry leading to GBS. These result suggest that these calf isolates are capable of initiating human infections. Although some of these *C. jejuni* had higher potential than others for invasion in epithelial cultures, we recognize that this is not a direct test of invasiveness in the human GI tract, yet it is a reasonable ethical alternative. As a control, the broth microdilution method showed that the minimum inhibitory concentration of gentamicin for each *C. jejuni* strain was as follows: 11168 (4.2 μg/ml), TW16491 (1.0 μg/ml), LM01 (2.1 μg/ml), LM08 (4.2 μg/ml), LM11 (4.2 μg/ml), LM12 (1.0 μg/ml), and LM24 (2.1 μg/ml). Because we used 250 μg/ml gentamicin in each well in the gentamicin killing assay, all of the *C. jejuni* strains used in this study were susceptible to killing by the antibiotic. Thus, we can attribute the *C. jejuni* isolated in the limiting dilution assay to cells that have entered the intracellular compartment.

**TABLE 6:** Comparison of invasion into Caco-2 intestinal epithelial cells of calf-derived and human-derived *Campylobacter jejuni* isolates with a variety of LOS types.

		Average cfu at final dilution (three replicates)		
*Campylobacter jejuni* Strain/origin/ST group	10E-1	1.00E-01	10E-3	10E-4
LM01 (calf – ST929)	TNTC	51	6	0
LM08 (calf – ST806)	TNTC	154	18	2
LM11 (calf – ST6227)	TNTC	115	11	1
LM12 (calf - ST922)	TNTC	37	6	0.3
LM24 (calf - ST982)	70	4	0	0
TW16491 (human - ST922)	10	0	0	0
Cj11168 (human)	264	21	3	0
Tissue Culture Medium	0	ND	ND	ND

*Limiting dilution assay results are given as average colony forming units at a specific dilution. TNTC, too numerous to count; ND, not done.*

## Discussion

Based on our results, the majority of the calf isolates belonged to LOS classes A, B, C, and E, which are most often associated with the development of GBS (Godschalk et al., 2007; Islam et al., 2009a,b). This result is consistent with previous studies of increased rates of neurological symptoms in farm workers. Two calf isolates and the isolate from the only family member colonized by *C. jejuni* had identical alleles for the seven MLST loci, *fla*A SVR, and *por*A as well as sharing antibiotic resistance loci; all three isolates belonged to LOS class E, indicating zoonotic transmission between family members and dairy calves. In addition, genome sequencing and analysis showed that 4 of 13 isolates carried a *cst*II allele containing an Asn51 substitution associated with GBS. Furthermore, all of the calf-derived strains were capable of invading human colonic epithelial cells. In fact, calf-derived isolates LM01, LM08, LM11, and LM12 invaded at higher rates than the positive invasive control C. *jejuni* 11168. Invasion potential existed in both LOS class E and A, B, C-typed strains associated with GBS diagnoses suggesting that any of these isolates could initiate a human infection. However, it should be noted that asymptomatic colonization with *C. jejuni* has been linked with development of GBS (St Charles et al., 2017). These data suggest that the family members were at risk of developing GBS.

*Campylobacter jejuni* is the leading cause of bacterial gastrointestinal infections and is commonly found in the gastrointestinal tracts of farm animals, including chickens and cattle (Vegosen et al., 2012). *C. jejuni* is the most common antecedent infection to the development of the autoimmune muscular neuropathy, GBS. Studies have been performed that show an increase in neurological symptoms in people who work with, or handle farm animals compared to people without this continuous exposure (Price et al., 2007; Vegosen et al., 2012). Previous studies have also demonstrated that *C. jejuni* is prevalent in a wide range of 0–51.2% of dairy farms in the U.S. (Harvey et al., 2004; Sanad et al., 2013). Therefore, when a small outbreak of *C. jejuni* occurred in humans living on a dairy farm in southwest Michigan, we not only characterized the isolates to determine whether zoonotic transmission had occurred between the dairy cattle and any family members residing on the farm, but also to determine the genetic relationships among the isolates. These findings–that the human isolate was virtually identical to two calf isolates in MLST sequence type, LOS type, antibiotic resistance profile, and genome content–indicate a high likelihood of interspecies transmission and suggest that dairy calves are an important reservoir for *C. jejuni* strains having LOS locus types associated with human infections and GBS.

Analysis of 12 *C. jejuni* calf isolates and the single human isolate by MLST loci, *fla*A SVR sequences, *por*A sequences, LOS classification, antibiotic resistance gene profiling, and protein identity comparison of unambiguously identified ORFs in whole-genome sequences showed that two calf isolates (LM03 and LM12) shared the same MLST sequence type (ST922), *fla*A allele (allele 81), *por*A allele (allele 2264), LOS class (E), antibiotic resistance gene pattern (beta-lactam and tetracycline but not aminoglycoside resistance) as the human isolate (TW16491). Furthermore, only eight of 1270 ORFs having unambiguously identified functions showed evidence of sequence divergence between isolates LM03, LM12, and TW16491. Based on the identity of results of all comparison methods for calf isolate LM12 and human isolate TW16491, we concluded that transmission was likely between the calf and the child; however, it is impossible to know the direction of transmission and to exclude the possibility that the family member infected the calf. Recall that four of the five STs in this small sample have been reported in contemporary human isolates from Michigan (Cha et al., 2016, 2017). This circumstance, in addition to the prevalence of these sequence types in Michigan cattle and the complex ecological and evolutionary relationships between highly mutable and recombining campylobacters in wild birds and animals common in farm environments (Sheppard et al., 2011a; Woodcock et al., 2017; Mourkas et al., 2020) also makes it impossible to ascertain the source of infection for the calves. Sheppard et al. (2011a) have noted that while host specificity can be observed in campylobacters carried by wild birds, the agricultural environment appears to promote “generalist” strains capable of colonizing several host species. In any case, there is strong evidence here suggesting that zoonotic transmission between dairy calves and family members occurred and may explain the frequent illnesses reported by the family.

Our results are similar to those obtained in other studies of *C. jejuni* in dairy and beef cattle. For example, a study done by Sanad et al. (2013) on a dairy farm in Ohio showed that 36.6% of the cattle were positive for *C. jejuni*. In that study the authors used MLST to examine the genetic relationships of the *C. jejuni* isolates found in both dairy cattle and starling birds and found the most common clonal complexes in the cattle to be CC21, CC42, CC45, and CC62 (Sanad et al., 2013). In a Finnish study, the authors performed MLST on 102 bovine *C. jejuni* isolates and found that 51% of the isolates belonged to clonal complex 21 (de Haan et al., 2010). Dingle et al. (2001) determined that clonal complexes CC21 and CC45 were the most common clonal complexes among human *C. jejuni* infections.

Jay-Russell et al. (2013) utilized *por*A sequencing and MLST sequences of human *C. jejuni* isolates to isolates taken from dairy cattle to study a milk-borne outbreak of campylobacterosis. These authors concluded that the level of strain discrimination provided by *por*A typing was very useful in identifying an association of dairy farm *C. jejuni* isolates with the milk-borne outbreak strain. In addition, CC21 was the most prevalent clonal complex among the isolates analyzed in that study (Jay-Russell et al., 2013).

Clonal complex ST-21 accounted for half of the isolates in our study (STs 806, 982, and 6227). Four of the five STs represented in our small sample (STs 806, 922, 929, and 982) were also detected in a larger survey of *C. jejuni* isolates from dairy and beef cattle in three Michigan herds; isolates of these four sequence types have also been recovered from human clinical samples in Michigan (Cha et al., 2016, 2017). The *C. jejuni* isolates in our study were isolated from a small set of calves from a single breeder and probably reflect a larger *C. jejuni* population in the dairy herd. Consistent detection of populations of multiple *C. jejuni* MLST types in previous studies of numerous beef and dairy herds and our finding of gene content variability in the complex loci encoding LOS and capsule biosynthesis and O-linked protein glycosylation taken together suggest that diversifying selection likely plays a role in maintaining *C. jejuni* population structure in these herds. The striking divergence in gene content among the ST806 calf isolates studied here also suggests the occurrence of horizontal gene transfer between *C. jejuni* strains within either the source population or the calf population. Such recombination has been well documented in *C. jejuni* populations (Fearnhead et al., 2005; Wilson et al., 2009; Biggs et al., 2011; Sheppard et al., 2011b,^2013^; Yahara et al., 2014; Samarth and Kwon, 2020). Its presence suggests added risk for transfer of AMR genes to humans and animals under these cattle management conditions.

Genome sequencing showed that both the general gene content and the virulence-associated gene content of strains of calf and the human isolates of ST922 were similar within that ST, as were those of the three ST929 and the two ST6227 isolates. The single ST982 isolate resembled the ST6227 isolates. Two of the ST806 isolates, LM08 and LM10, resembled each other while LM13 and LM26 diverged in differing degrees, possibly due to recombination. All calf and human isolates reported here clustered strongly with clinical strains 11168, CF93-6, and 84-25 in virulence-associated gene content. As expected, the greatest variation in virulence-associated gene content lay in the complex loci encoding *C. jejuni* cell surface molecules, while the presence or absence of ORFs homologous to the remainder of virulence-associated genes was remarkably consistent across all isolates. We conclude that all isolates reported here are potentially capable of producing disease in humans.

Finally, the high incidence of isolates carrying LOS locus classes and HS Penner serotypes associated with risk of GBS even in this small sample justify larger studies in US dairy herds, especially given the growing popularity of herd share arrangements and raw milk consumption.

## Data Availability Statement

The datasets presented in this study can be found in online repositories. The names of the repository/repositories and accession number(s) can be found in the article/(Table 1) Supplementary Material.

## Ethics Statement

The studies involving human participants were reviewed and all protocols were approved by the Institutional Review Boards at Michigan State University (IRB# 10-736SM) and the Michigan Department of Health and Human Services (MDHHS) (842-PHALAB). Written informed consent to participate in this study was provided by the participants’ legal guardian/next of kin.

## Author Contributions

JS isolated *Campylobacter* strains, conducted multiplex PCR species identification, MSLT, *fla*A, *por*A, and LOS PCR/RFLP typing of isolates, and wrote the manuscript, which comprised a portion of her doctoral dissertation. PB conducted whole genome sequencing and conducted genomic analysis using PATRIC and RAST. JB analyzed whole genome data, conducted phylogenetic analyses, assisted with laboratory methods and wrote the manuscript. HA and MV conducted the gentamicin killing assays and assisted with the bioinformatic analyses. SM provided training of JS for the MLST analyses, coordinated the acquisition of the human samples and analyzed them or provided them for the study. LM made the contact with the referring veterinarian to initiate the on-farm study, arranged for samples to be sent to MSU from the referring veterinarian and continued this communication, provided funding and oversight of the project, analyzed the data, assisted with gentamicin killing assay and wrote the manuscript. All the authors contributed to the article and approved the submitted version.

## Conflict of Interest

The authors declare that the research was conducted in the absence of any commercial or financial relationships that could be construed as a potential conflict of interest.

## Publisher’s Note

All claims expressed in this article are solely those of the authors and do not necessarily represent those of their affiliated organizations, or those of the publisher, the editors and the reviewers. Any product that may be evaluated in this article, or claim that may be made by its manufacturer, is not guaranteed or endorsed by the publisher.

## Footnotes

1
http://pubmlst.org/campylobacter/info/porA_method.shtml

2
http://pubmlst.org/campylobacter/

3
https://www.ebi.ac.uk/Tools/msa/clustalo/

4
https://pubmlst.org/

## Supplementary Material

Supplementary Table 1 Presence/absence of 55 LOS open reading frames in calf isolates. This table contains contiguous homologs in each isolate to the 55 LOS loci detailed in Parker et al. (2005, 2008) and Richards et al. (2013). Homologs were identified by conducting TBLASTN of individual reference sequences to the genome of each isolate. *E* values ≤ 1 × 10^–30^ are reported as positive hits; *E* values ≥ 1 × 10^–30^ are reported as “no match.” Only hits having the smallest E values (least likelihood of occurring by chance) are shown.

Supplementary Table 2 Protein percent identity of calf isolate open reading frames as determined in RAST. This figure shows the protein percent identity of open reading frames each strain in this study to the open reading frames of the reference strain LM01. Clinical *C. jejuni* isolates 260.94, HB93-13, CF93-6, 84-25, 81-176, and 11168, *C. jejuni* chicken isolate RM1221, *C. jejuni* subsp. *doylei* 269.97, and *C. coli* RM2228 are included. **(A)** Open reading frames with functions unambiguously identified in RAST; **(B)** open reading frames with probable, possible, or putative functions identified in RAST; **(C)** hypothetical proteins identified in RAST; **(D)** antibiotic resistance genes identified in RAST; **(E)** plasmid or bacteriophage associated open reading frames identified in RAST.

Supplementary Table 3 Protein percent identity of virulence-associated calf isolate open reading frames. Clinical *C. jejuni* isolates 260.94, HB93-13, CF93-6, 84-25, 81-176, and 11168 are included. This table contains homologs in each isolate to 342 virulence-associated *C. jejuni* loci detailed in Bell et al. (2013) and updated from the literature (Jacobs et al., 2008; Baek et al., 2011; Buelow et al., 2011; Barrero-Tobon and Hendrixson, 2012; Boehm et al., 2012; Cullen et al., 2012, 2013; Frirdich et al., 2012). Homologs were identified by conducting TBLASTN of reference sequences to the genome of each isolate. The reference sequences from GenBank that were employed for this analysis are given in column 1; sequence names of the form Cj####.gene_name are from *C. jejuni* NCTC 11168 genome sequence AL111168.
