# Supplementary figures and images for: Zoonotic Transmission of Campylobacter jejuni to Caretakers From Sick Pen Calves Carrying a Mixed Population of Strains With and Without Guillain Barré Syndrome-Associated Lipooligosaccharide Loci
Source: Front Microbiol. 2022 Apr 29;13:800269. doi: 10.3389/fmicb.2022.800269 (PMC9112162; doi:10.3389/fmicb.2022.800269)

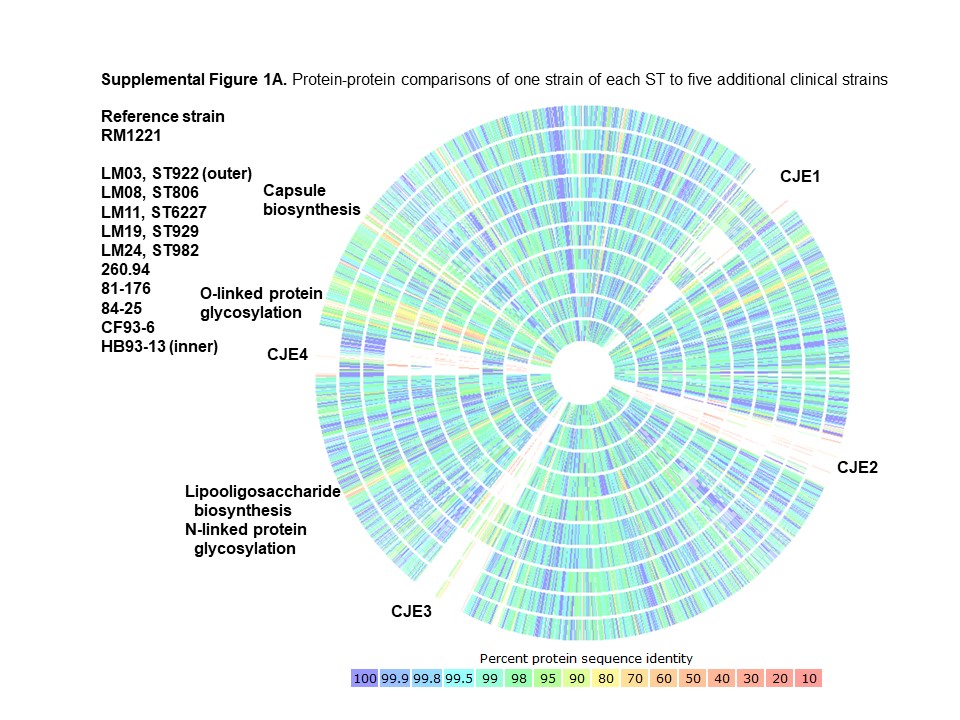

Supplement: Supplementary file 5 [file Image_1.JPEG]

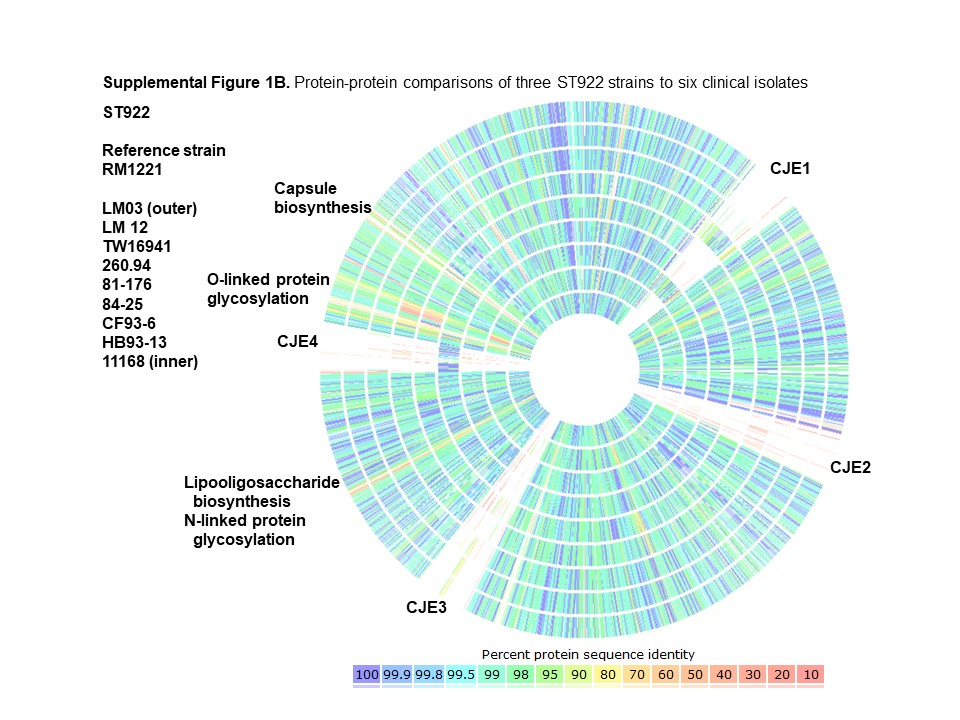

Supplement: Supplementary file 6 [file Image_2.JPEG]

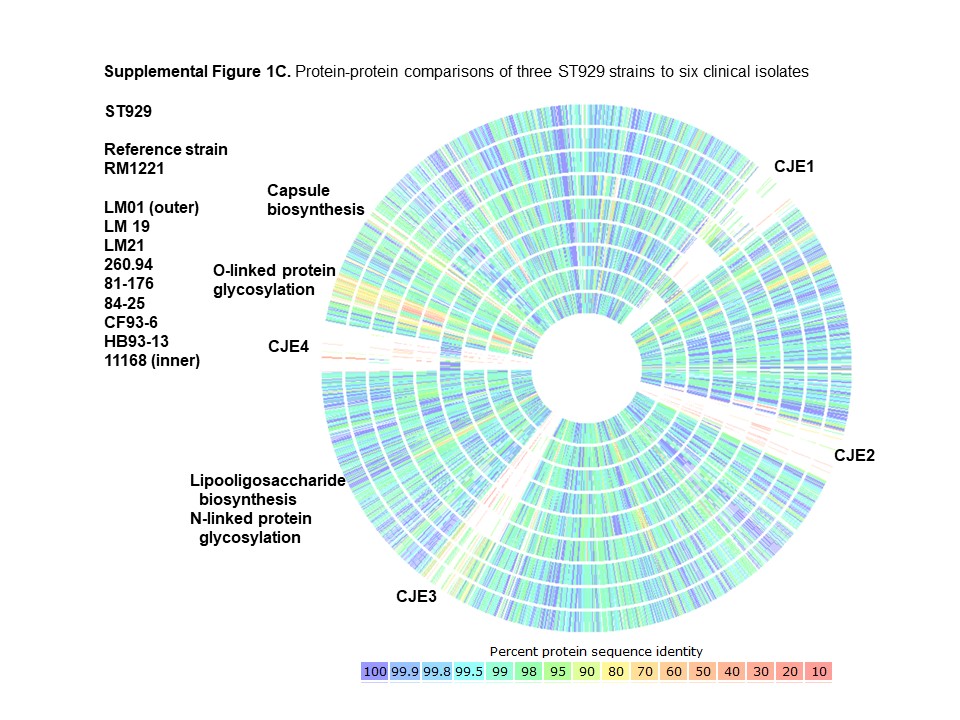

Supplement: Supplementary file 7 [file Image_3.JPEG]

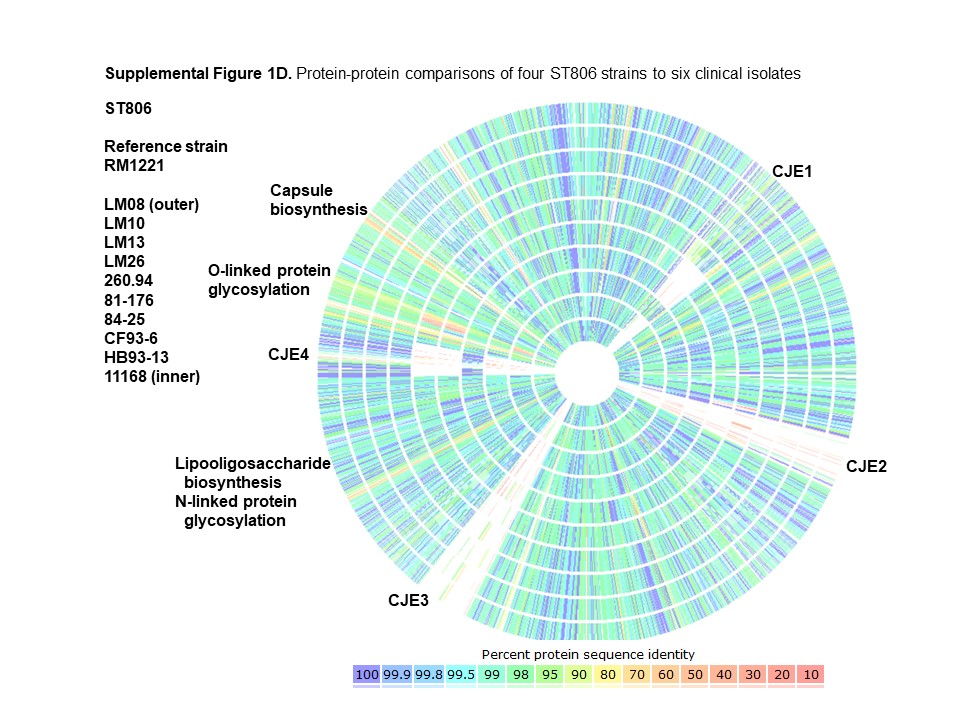

Supplement: Supplementary file 8 [file Image_4.JPEG]

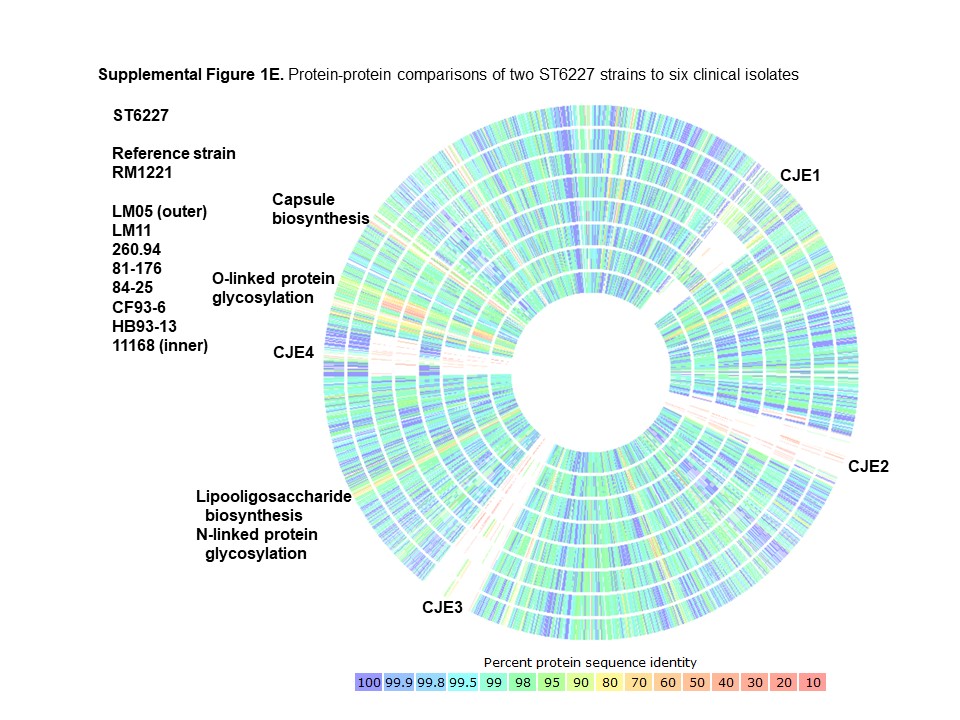

Supplement: Supplementary file 9 [file Image_5.JPEG]

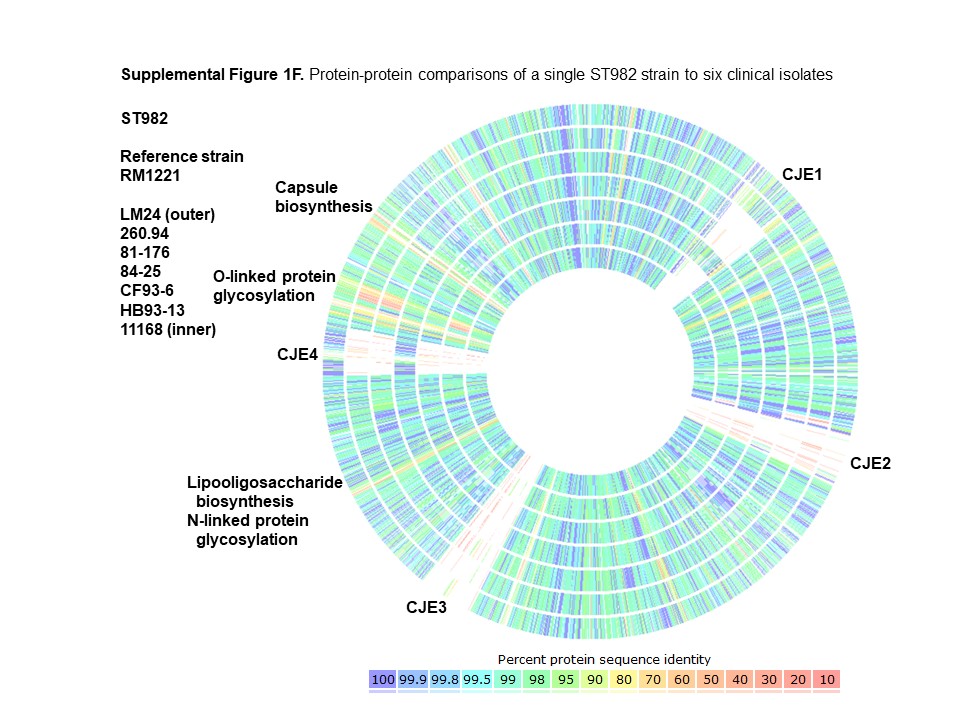

Supplement: Supplementary file 10 [file Image_6.JPEG]
